# Catalytic Advantages of SO_3_H-Modified UiO-66(Zr) Materials Obtained via Microwave Synthesis in Friedel–Crafts Acylation Reaction

**DOI:** 10.1021/acs.inorgchem.4c01792

**Published:** 2024-09-03

**Authors:** Marta Bauza, Pedro Leo, Carlos Palomino Cabello, Antonio Martin, Gisela Orcajo, Gemma Turnes Palomino, Fernando Martinez

**Affiliations:** † Department of Chemistry, University of the Balearic Islands, Cra. de Valldemossa, Palma de Mallorca 07122, Spain; ‡ Chemical and Environmental Engineering Group. ESCET, Universidad Rey Juan Carlos. c/Tulipán s/n, Móstoles 28933, Spain; § Instituto de Tecnologías para la Sostenibilidad. Universidad Rey Juan Carlos. C/Tulipán s/n, Móstoles 28933, Spain

## Abstract

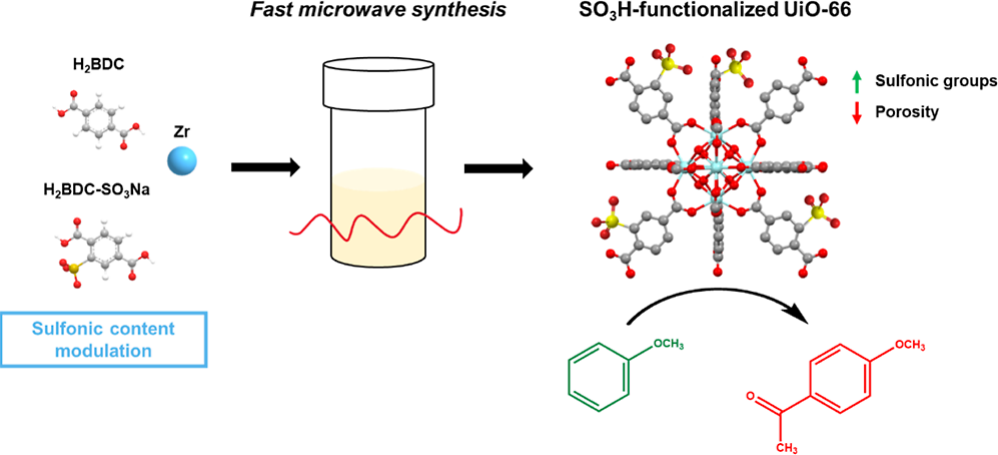

The catalytic activity and stability of sulfonic-based UiO-66(Zr) materials were tested in the Friedel–Crafts acylation of anisole with acetic anhydride. The materials were prepared using microwave-assisted synthesis, producing microporous materials with remarkable crystallinity and physicochemical features as acid catalysts. Different ratios between both organic ligands, terephthalic acid (H_2_BDC) and monosodium 2-sulfoterephthalic acid (H_2_BDC–SO_3_Na), were used for the synthesis to modulate the sulfonic content. The sulfonic-based UiO-66(Zr) material synthesized with a H_2_BDC/H_2_BDC–SO_3_Na molar ratio of 40/60 exhibited the best catalytic performance in the acidic-catalyzed Friedel–Crafts acylation reaction. This ratio balanced the number of sulfonic acid sites and their accessibility within the UiO-66 microporous structure. The catalytic performance of this material increased remarkably at 200 °C, outperforming reference acids and commercial heterogeneous catalysts such as Nafion-SAC-13 and Amberlyst-70. Additionally, the best sulfonic-based UiO-66(Zr) material proved to be stable in four successive reaction cycles, maintaining both its catalytic activity and its structural integrity.

## Abstract 2

Sulfonic-based UiO-66(Zr) materials, synthesized using microwave-assisted methods, show excellent catalytic activity and stability in Friedel–Crafts acylation of anisole with acetic anhydride, outperforming commercial catalysts.

## 1 Introduction

Friedel–Crafts acylation is one of the most critical reactions in synthesizing aromatic ketones, which are useful intermediates in producing numerous valuable fine chemical products such as fragrances, flavoring agents, or pharmaceutical components.^1−3^ This reaction is an electrophilic aromatic substitution produced through the reaction of aromatic substrates with an acyl group in the presence of a Lewis or a Brønsted acid catalyst. Between the aromatic compounds, the acylation of anisole is one of the most studied catalytic reactions by the scientific community, not only because of the importance of the products but also as a test reaction to evaluate the acidity/basicity of the catalysts.^4,5^ In recent years, many studies have been carried out using zeolites,^6−8^ heteropolyacids,^9−11^ or functionalized silica materials^12,13^ as heterogeneous catalysts for this reaction.

Metal–organic frameworks (MOFs) have emerged as novel heterogeneous catalysts for different reactions by taking advantage of their regular frameworks and the great variety in their chemical nature.^14−16^ The use of MOFs as acid catalysts for anisole acylation is not very common, but some scarce examples demonstrate their unequivocal viability.^17^ In 2014, Jiang and co-workers^18^ reported a high catalytic activity of the sulfated MOF-808 in anisole acylation reactions with different acylation reagents, but the material stability and reutilization were not evaluated. Not long after, Khder and co-workers^19^ published the employment of 12-tungstophosphoric acid-supported MIL-101 following an impregnation method. In this last study, a slight loss of catalytic activity was observed with the increase in the number of reaction cycles, probably due to the strong adsorption of anisole acylation reaction products over the acid sites of the catalyst. In a later study, 2017, a Brønsted acidic ionic liquid, *N*-methyl-2-pyrrolidonium methyl sulfonate, was immobilized on the MIL-101 material by an impregnation method.^20^ The functionalized MOF obtained showed remarkable anisole conversion, but the reusability of this reaction was not tested. Recently, we have published the potential application of sulfonic acid-functionalized UiO-66(Zr) and MIL-101(Cr) materials, prepared by direct synthesis with a sulfonic acid-containing benzene dicarboxylate linker, as heterogeneous catalysts in the Friedel–Crafts acylation reaction of anisole using acetic anhydride as an acylation agent.^21^ The MIL-101-SO_3_H material achieved a slightly better performance regarding anisole conversion than those obtained for the reference commercial perfluorosulfonic acid-based catalyst, Nafion-SAC-13. Moreover, this material was successfully regenerated by a sustainable method for removing reagents and polyacetylated products, maintaining its catalytic activity and crystalline structure after four successive reaction cycles of 5 h, in contrast to the Nafion-SAC-13 material, which was deactivated entirely after the first reaction cycle. This work also reported a low anisole conversion for the sulfonic UiO-66(Zr) material due to the reduced porosity and limited accessibility to the sulfonic acid sites.

One of the most important limitations for the commercial application of MOFs as catalysts is that the majority of their preparation is based on solvothermal methods, which entail heating to the reaction temperature, longer synthesis times, and lower yields. In this context, microwave-assisted synthesis is one of the best alternatives to conventional solvothermal synthesis since it can increase the synthesis yields using lower reaction times with fine control of the morphology and size of the crystals obtained.^22,23^ Through this approach, different MOFs, such as MOF-74,^24^ MIL-125,^25^ MOF-5,^26^ and MIL-101,^27^ among others, have been successfully synthesized.

The present work deals with the catalytic study of SO_3_H-functionalized UiO-66(Zr) MOFs with different contents of sulfonic groups to evaluate the accessibility of their acid sites in the Friedel–Crafts acylation of anisole with acetic anhydride. The acylating agent has been selected because it is one of the agents with lower catalytic activity,^28^ demonstrating the materials’ catalytic capacity even under the least favorable conditions. The SO_3_H-modified UiO-66(Zr) materials were prepared by an easy and fast microwave-assisted method using different ratios of terephthalic acid and monosodium 2-sulfoterephthalic acid as organic ligands, obtaining different acidic microporous materials. The influence of including sulfonic acid groups on the textural properties, accessibility to the acid sites of the materials, and potential catalytic activity will be evaluated. Optimal catalytic reaction conditions will also be assessed for the best-synthesized SO_3_H-modified UiO-66(Zr) material and its reusability in consecutive reaction cycles.

## 2 Experimental Section

### 2.1 Chemicals

*N*,*N*′-Dimethylacetamide (DMA, ≥99.5%) and ethanol (≥99.8%) were obtained from Scharlab. Monosodium 2-sulfoterephthalic acid (H_2_BDC–SO_3_Na, > 98%) was procured from the Tokyo Chemical Industry. Zirconyl chloride octahydrate (ZrOCl_2_·8H_2_O, ≥ 99%) was acquired from Merck. Terephthalic acid (H_2_BDC, ≥ 99%), formic acid (≥99%), anisole, and acetic anhydride were obtained from Sigma-Aldrich. The Nafion-SAC-13 catalyst was also supplied by Sigma-Aldrich. The Amberlyst 70 catalyst was obtained from Rohm and Haas and used without further treatment.

### 2.2 Synthesis of SO_3_H-Modified UiO-66(Zr) Materials

Different sulfonic-functionalized UiO-66(Zr) MOFs were prepared, varying the molar ratio between H_2_BDC and H_2_BDC–SO_3_Na linkers by a rapid microwave-assisted method adapted from a conventional solvothermal procedure previously reported.^29^ Typically, a solution of 3.1 mmol of ZrOCl_2_·8H_2_O, 3.1 mmol of the organic linkers (H_2_BDC–SO_3_Na and H_2_BDC alone or mixtures with different H_2_BDC–SO_3_Na/H_2_BDC molar ratios), and 11.7 mL of formic acid in 40 mL of DMA was stirred for 30 min at room temperature. After that, the resulting mixture was transferred to a Teflon vessel and heated at 150 °C for 2 h in a microwave oven (Start D, Milestone maximum power 350 W). Finally, the solid was collected by centrifugation, washed thoroughly with ethanol and acetone, and dried under vacuum. Sulfonic-containing UiO-66(Zr) materials with H_2_BDC–SO_3_Na/H_2_BDC molar ratios of 0/100, 20/80, 40/60, 60/40, 80/20, and 100/0 were denoted as UiO-66, (20)SO_3_H-UiO-66, (40)SO_3_H-UiO-66, (60)SO_3_H-UiO-66, (80)SO_3_H-UiO-66, and (100)SO_3_H-UiO-66, respectively. All the materials were activated under vacuum at 150 °C for five h before being used in the catalytic reaction.

### 2.3 Characterization

X-ray diffraction (XRD) patterns were obtained by using Cu Kα radiation on a Bruker D8 Advance diffractometer. Nitrogen adsorption isotherms were measured at 77 K using a TriStar II (Micromeritics) gas adsorption analyzer. The samples were previously outgassed at 150 °C overnight. The isotherms were analyzed using the Brunauer–Emmett–Teller (BET) method to determine the specific surface area and the two-dimensional nonlocal density functional theory model (2D-NLDFT) to obtain the pore size distribution. Thermogravimetric analysis (TGA) was conducted in a nitrogen atmosphere using a TA Instrument SDT 2960 simultaneous DSC-TGA. Energy-dispersive X-ray spectroscopy (EDS) spectra were acquired using a scanning electron microscope, Hitachi S-3400N, equipped with a Bruker AXS XFlash 4010 energy-dispersive X-ray spectroscopy system. Electron micrographs were obtained using a transmission electron microscope Thermo Scientific Talos F200i operated at 80 kV. Elemental C, H, N, and S analyses were carried out with a PerkinElmer 240C elemental analyzer. FTIR spectra were acquired by using a Bruker Tensor 27 spectrometer equipped with the ATR platinum module. The acidity of the samples was evaluated through CD_3_CN adsorption at room temperature, followed by FTIR spectroscopy using a Bruker Vertex 80v spectrophotometer operating at 3 cm^–1^ resolution. For that, a self-supporting MOF wafer was prepared and degassed inside an IR cell under dynamic vacuum at 423 K for 8 h.

### 2.4 Reaction Procedure

The catalytic performance of UiO-66(Zr) materials with different contents of sulfonic acid groups was evaluated in the acylation of anisole with acetic anhydride to form o- and *p*-methoxyacetophenones (MAPs) as a reference acid-catalyzed reaction. The catalytic experiments were conducted in a round-bottomed flask under a N_2_ atmosphere. Reactants and the catalyst were charged at room temperature and then heated to reaction temperature using a silicone bath. Typically, an equimolar anisole/acetic anhydride molar ratio without solvent and a catalyst concentration of 1.25 wt % to the anisole mass are mixed at 500 rpm of stirring speed to avoid diffusional mass transfer limitations. No solvent is chosen to prevent the known environmental drawbacks of using organic solvents. Aliquots were withdrawn at selected reaction times of 1 and 5 h. Anisole and o- and p-MAPs were quantified by gas chromatography using a GC-3900 Varian chromatograph equipped with a DB-5MS Ultra Inert column (30 m × 0.25 mm, film thickness 0.25 μm) and a flame ionization detector. Sulfolane was used as an internal standard, and all samples were analyzed three times. A comparative analysis was conducted between the most efficient UiO-66 catalyst with sulfonic groups and the commercial catalysts. The analysis was carried out by adding a proportion of 1.25 wt % with respect to the initial mass of anisole and maintaining a temperature of 200 °C. The most efficient UiO-66 catalyst with sulfonic groups was also subjected to reuse under the aforementioned reaction conditions. However, between each catalytic cycle, the catalyst was subjected to a regeneration process to ensure the effective elimination of the polyacyl products generated during the reaction.

## 3 Results and Discussion

### 3.1 Characterization of Sulfonic-Containing UiO-66(Zr) Materials

Table 1 shows the synthesis yield, textural properties, and sulfur content of microwave-assisted synthesized sulfonic-containing UiO-66(Zr) materials. Regarding previous experiments, 2 h microwave-assisted synthesis was fixed for all materials since the high yield was obtained. The synthesis yield reached remarkable values of ca. 90% for the H_2_BDC–SO_3_Na/H_2_BDC molar ratio between 20/80 ((20)SO_3_H-UiO-66) and 60/40 ((60)SO_3_H-UiO-66). These yields were slightly higher than those of UiO-66(Zr) without the sulfonic-containing linker. However, the increase of the H_2_BDC–SO_3_Na/H_2_BDC molar ratio above 60/40 significantly reduced the synthesis yield, leading to a value of 52% when only the H_2_BDC–SO_3_Na linker was used ((100)SO_3_H-UiO-66). These results indicate a lower ability of the sulfonic-containing linker to coordinate the zirconium cations when only this organic ligand is employed.

**Table 1:** Physicochemical Properties of Sulfonic-Containing UiO-66(Zr) Materials

catalyst	BET (m^2^·g^–1^)^a^	S_micro_ (m^2^·g^–1^)^b^	S_Ext_ (m^2^·g^–1^)^b^	V_p_ (cm^3^·g^–^1)^c^	synthesis yield (%)^d^	(mmol S/g)^e^	formula^f^
UiO-66	908	812	95	0.48	79		Zr_6_O_7.3_(BDC)_4.7_
(20)SO_3_H-UiO-66	703	633	70	0.37	89	0.39	Zr_6_O_7.4_(BDC)_4_(SO_3_H-BDC)_0.6_
(40)SO_3_H-UiO-66	650	588	62	0.34	88	0.79	Zr_6_O_7.5_(BDC)_3.2_(SO_3_H-BDC)_1.3_
(60)SO_3_H-UiO-66	446	356	90	0.32	90	1.04	Zr_6_O_8.2_(BDC)_2.1_(SO_3_H-BDC)_1.7_
(80)SO_3_H-UiO-66	290	252	38	0.17	83	1.30	Zr_6_O_8.7_(BDC)_1.3_(SO_3_H-BDC)_2_
(100)SO_3_H-UiO-66	250	182	68	0.22	52	1.49	Zr_6_O_8.9_(SO_3_H-BDC)_3.1_

a Total surface area calculated using the BET method from the adsorption branch of the corresponding nitrogen isotherm.

b Micropore and external surface area calculated using the t-plot method.

c Total pore volume recorded at *P*/*P*_o_ = 0.95.

d Synthesis yield based on the linker content.

e Sulfur content calculated from elemental analysis.

f Dehydroxylated chemical formula determined from elemental analysis and TGA in air.

**Figure 1:**
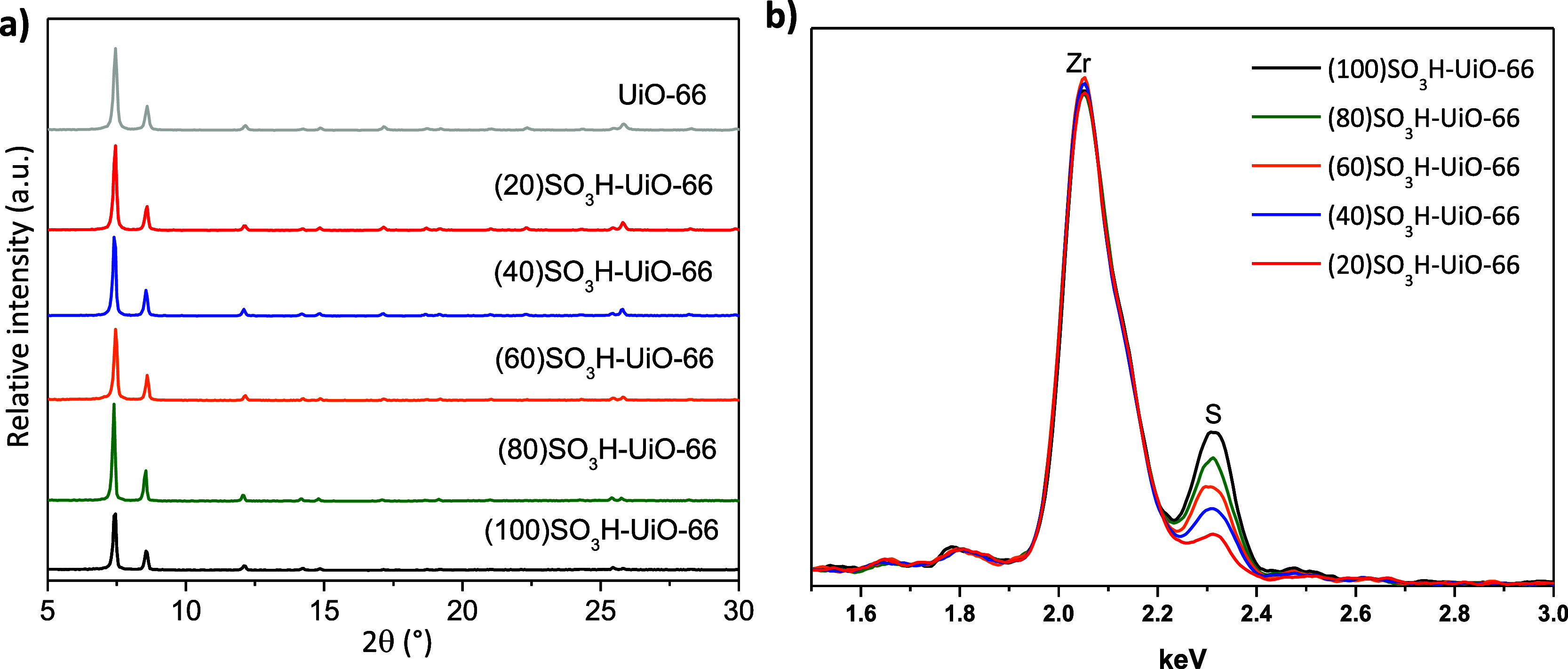
(a) XRD patterns and (b) EDS spectra of UiO-66(Zr) and sulfonic UiO-66(Zr) materials.

Figure 1a shows the XRD patterns of UiO-66(Zr) materials with different amounts of sulfonic groups. All the diffractograms showed the characteristic XRD pattern previously reported for the UiO-66 structure.^30^ The presence of sulfonic acid groups in the UiO-66(Zr) samples was observed by EDS spectroscopy (Figure 1b). The EDS spectrum of all SO_3_H-UiO-66 catalysts shows two bands at 2.05 and 2.31 keV, corresponding to the Zr (L_α_) and S (K_α_) peaks, respectively. It is also worth noting that the intensity of the S (K_α_) peak increases as the H_2_BDC–SO_3_Na/H_2_BDC ratio rises, indicating an increase in the number of sulfonic groups from the (20)SO_3_H-UiO-66 to the (100)SO_3_H-UiO-66 samples, which also agrees with the elemental analysis of the SO_3_H-UiO-66 samples (Table 1). TGA was carried out to evaluate the thermal stability of the prepared samples (Figure S1). The TGA curves showed one weight loss step in the 30–100 °C temperature range, attributed to the removal of physically adsorbed water molecules in the materials, and a second weight loss up to 210 °C, which may be related to the removal of occluded DMA solvent inside the pores and the dihydroxylation of the metal-oxo cluster. The temperature of the final weight loss, due to the complete framework decomposition, decreases as the amount of sulfonic acid content increases, going from 475 °C (UiO-66) to 390 °C ((100)SO_3_H-UiO-66). The chemical formulas and defect concentrations of the UiO-66(Zr) samples were approximately determined by combining the sulfur content calculated from elemental analysis with the aerobic decomposition data from TGA (Table 1). According to the quantitative calculation method used in the literature,^31,32^ which compares the TGA plateau at 400 °C (solvent-free and dehydrated materials) and the end weight at 800 °C (Figure S2), the number of defects in samples UiO-66(Zr), (20)SO_3_H-UiO-66, (40)SO_3_H-UiO-66, (60)SO_3_H-UiO-66, (80)SO_3_H-UiO-66, and (100)SO_3_H-UiO-66 was 1.3, 1.4, 1.5, 2.2, 2.7, and 2.9, respectively, indicating that as the proportion of sulfonic groups increases, the number of defects also increases.

The set of sulfonic-containing UiO-66(Zr) materials was also characterized by FTIR spectroscopy (Figure S3). The FTIR spectrum of UiO-66 exhibited absorption bands at 1550–1630 and 1450–1580 cm^–1^, which correspond to C=O in carboxylates and C=C in aromatic compounds, respectively, matching well with those reported in the literature for the UiO-66 structure.^33^ The sulfonic-containing UiO-66(Zr) materials showed additional peaks with increasing intensity from (20)SO_3_H-UiO-66 to (100)SO_3_H-UiO-66 samples. The peak at 1224 cm^–1^ is attributed to the stretching modes O=S=O, and the peaks at 1070 and 617 cm^–1^ are assigned to the stretching modes S–O and C–S, respectively.^34−37^

**Figure 2:**
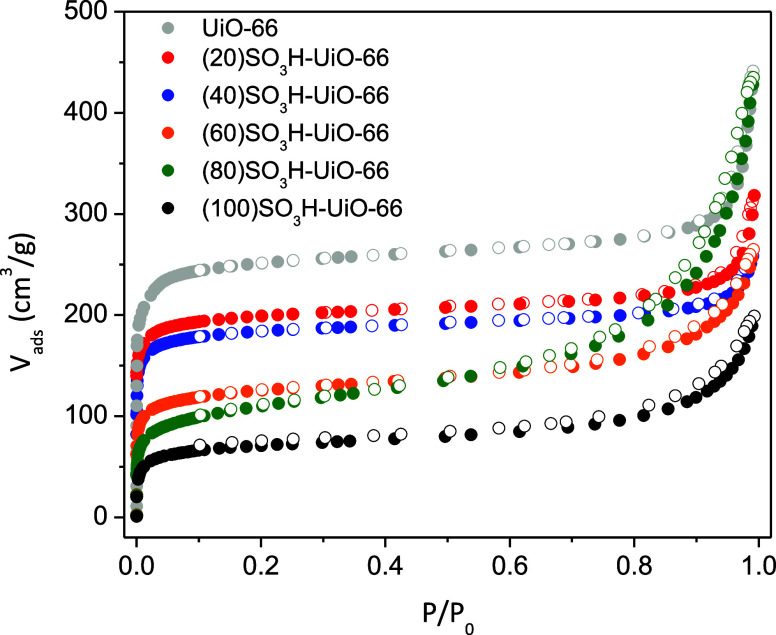
N_2_ adsorption–desorption isotherms of UiO-66(Zr) and sulfonic UiO-66(Zr) materials.

All the sulfonic-containing UiO-66(Zr) materials exhibited a significant nitrogen uptake at a low relative pressure (*P*/*P*_0_ < 0.02) of the nitrogen adsorption–desorption isotherms (Figure 2). The BET surface area varied from 703 to 250 m^2^/g and the total pore volume from 0.37 to 0.17 cm^3^/g for the (20)SO_3_H-UiO-66 to (100)SO_3_H-UiO-66 materials, values which were lower than those of the pristine UiO-66(Zr) material (Table 1). The textural properties of UiO-66(Zr) (Table 1) agree with previous results of this material synthesized using conventional methods.^30^ The BET surface area of sulfonic-enriched UiO-66(Zr) materials is also mainly due to the micropore surface area, and it decreased as the H_2_BDC–SO_3_Na/H_2_BDC molar ratio increased, probably due to a partial occupation of the space inside the pores by the sulfonic acid groups. This reduction in textural properties has been observed in other MOF materials postsynthesis functionalized with sulfonic groups, such as MIL-101-Cr-NH_2_, where the surface area was reduced by around 70% compared to the pristine MOF material.^38^ Regarding the pore size distribution, all of the UiO-66(Zr) samples display similar pore widths centered around 10 Å (Figure S4).

### 3.2 Evaluation of Catalytic Activity

The catalytic activity of the sulfonic-containing UiO-66(Zr) materials synthesized with different H_2_BDC–SO_3_Na/H_2_BDC molar ratios was evaluated in the acylation of anisole with acetic anhydride to obtain methoxyacetophenones (MAPs). The study also examined the impact of reaction temperature on the catalytic activity and stability of sulfonic-containing UiO-66(Zr) materials, comparing them to the reference commercial catalysts Nafion-SAC-13 and Amberlyst-70. Finally, the reusability of the best-synthesized material was evaluated for four consecutive cycles.

#### 3.2.1 Influence of the H_2_BDC–SO_3_Na/H_2_BDC Molar Ratio

**Figure 3:**
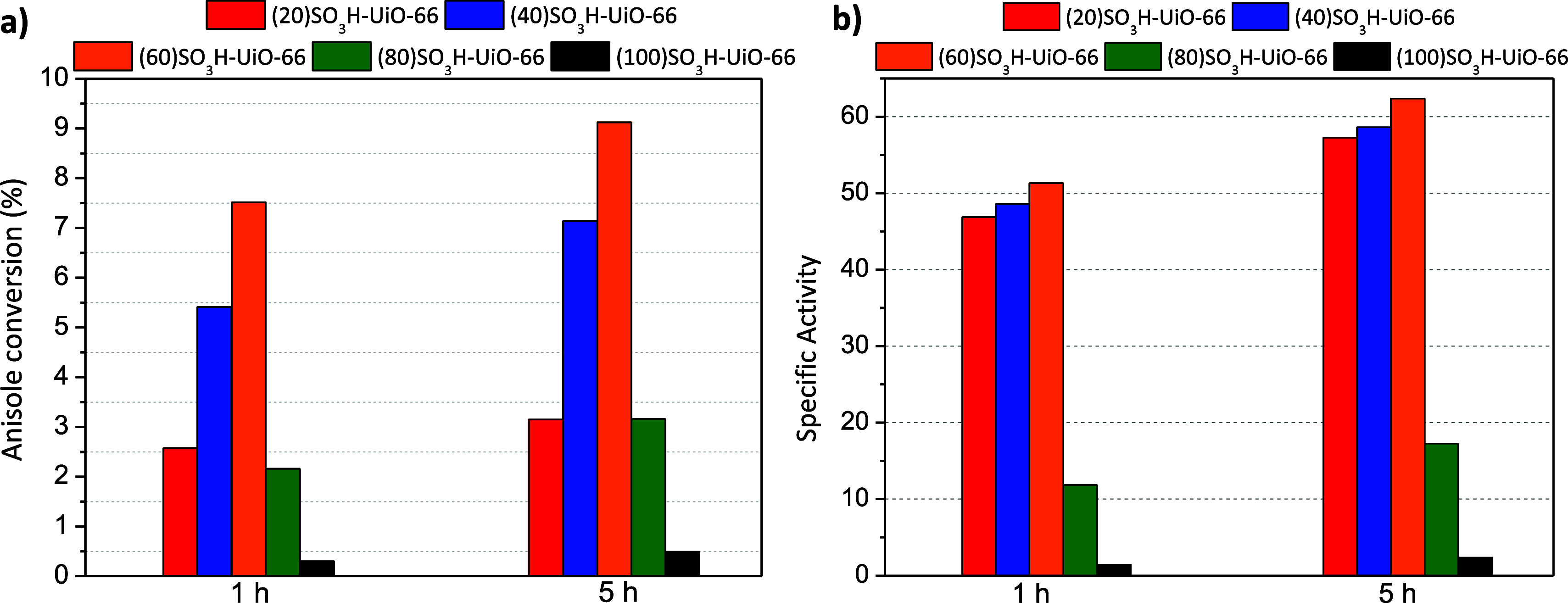
Anisole conversion (a) and specific activity (b) of sulfonic UiO-66(Zr) materials.

The catalytic performance of sulfonic-containing UiO-66(Zr) materials was assessed in terms of anisole conversion and p-MAP selectivity, and specific activity was calculated as mmol of reacted anisole per mmol of S at 1 and 5 h of reaction time (Figure 3). These experiments were performed at 150 °C with an equimolar anisole/acetic anhydride molar ratio without solvent and 1.25 wt % of the catalyst to anisole mass. These reaction conditions were selected based on our previous work.^21^ The anisole conversion results demonstrated that the UiO-66(Zr) material was inactive due to the absence of active sulfonic acid groups (not shown). It is known that the presence of strong Brønsted acid sites plays an important role in Friedel–Craft reactions.^2^ Within the SO_3_H-UiO-66 series of samples, the best anisole conversion was achieved for the (60)SO_3_H-UiO-66 material (Figure 3a). The catalytic activity of the sulfonic UiO-66(Zr) materials showed two different behaviors. The samples with a smaller number of sulfonic groups (20, 40, and 60-SO_3_H-UiO-66 materials) displayed increased catalytic activity proportional to the number of acid centers (Table 1). However, in the case of 80 and 100-SO_3_H-UiO-66 materials, the anisole conversion dramatically decreased, despite having a higher number of sulfonic acid centers. These results showed a lower availability of the sulfonic acid sites in samples with higher sulfonic groups. To check this hypothesis, the specific activity of the materials per millimole of S was also assessed (Figure 3b). As can be seen, the specific activity is practically constant for the three materials with fewer sulfonic groups, confirming the total accessibility of their sulfonic acid sites. On the other hand, a remarkable reduction of the specific activity was observed for samples 80 and 100-SO_3_H-UiO-66 because the sulfonic groups are partially blocked, as can also be deduced from the reduction of BET surface area (Table 1). The findings demonstrated that the catalytic activity is not contingent upon the number of defects present in the materials. Additionally, it must also be pointed out that all the materials displayed a drastic decrease in the reaction rate between the starting point and the first hour of the reaction compared to the interval between 1 and 5 h of reaction. This behavior can be attributed to the catalyst deactivation that classically happens in these acid-catalyzed acylation reactions.^39^ Moreover, all the catalysts showed a high selectivity toward the p-MAP product (over 92%, not shown).

After confirming that (60)SO_3_H-UiO-66 shows the highest anisole conversion value, a comparison was made between this material and a similar material but synthesized under solvothermal conditions (150 °C for 24 h; Figure S5) to evaluate the influence of the synthesis procedure in the catalytic performance. Although the solvothermal material exhibits superior textural properties and a smaller crystal size (see Figures S6 and S7, respectively) compared to the microwave-synthesized material, its catalytic behavior was similar. The solvothermal material showed a slight increase of 0.5 and 0.2% in anisole conversion value after 1 and 5 h of reaction, respectively. These results indicate that the method of catalyst synthesis does not significantly affect the catalytic performance of the reaction, confirming that microwave-assisted synthesis is a reproducible and cost-effective method for producing high-quality MOF catalysts in an efficient and sustainable procedure.^40,41^

#### 3.2.2 Influence of the Reaction Temperature

**Figure 4:**
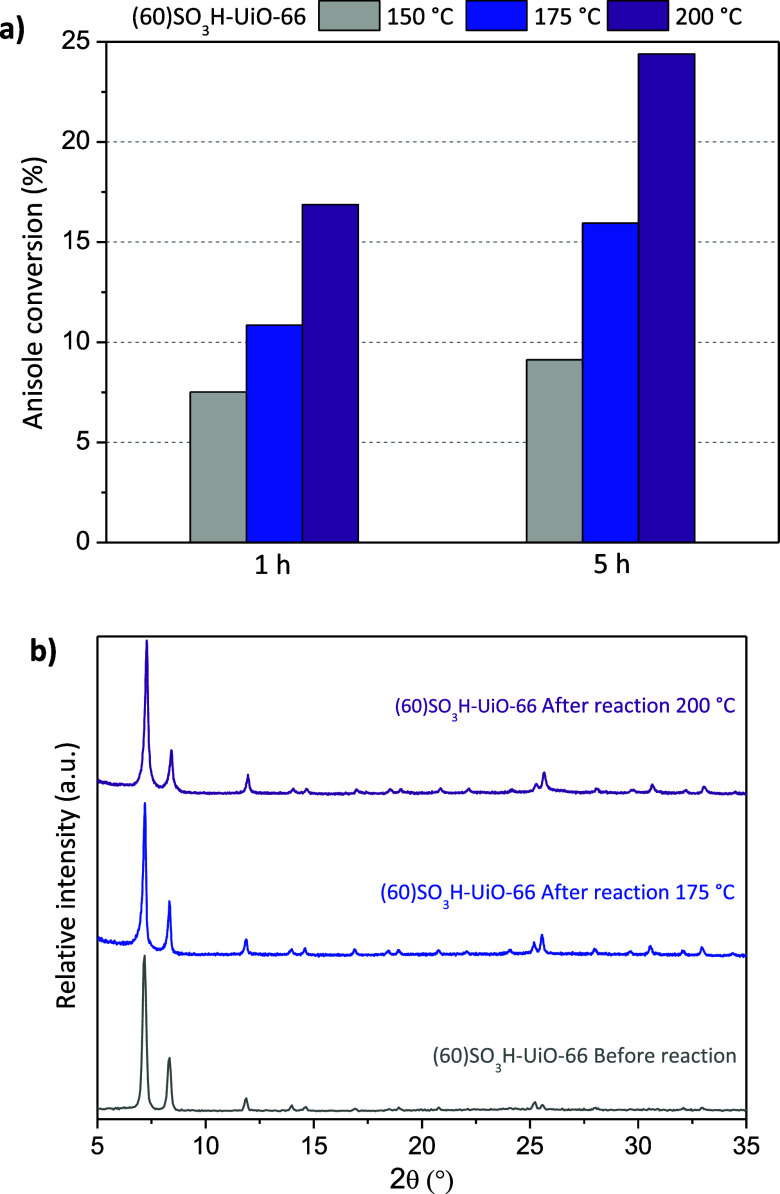
(a) Influence of temperature on the catalytic activity of the (60)SO_3_H-UiO-66 catalyst and (b) XRD of the (60)SO_3_H-UiO-66 catalyst after 5 h of reaction at different temperatures.

Previous works have demonstrated a remarkable enhancement of anisole conversion when the temperature increases.^39,42^ Thus, the catalytic activity and stability of the most active (60)SO_3_H-UiO-66 material were evaluated at 150, 175, and 200 °C using an equimolar anisole/acetic anhydride molar ratio without solvent and 1.25 wt % of catalyst with respect to initial anisole mass. Figure 4 shows the catalytic performance and XRD patterns of the recovered (60)SO_3_H-UiO-66 material after the reaction. The temperature increase from 150 to 200 °C enhanced the catalytic performance of the (60)SO_3_H-UiO-66 material. A remarkable conversion of 17% of anisole was achieved after 1 h and 24% after 5 h at 200 °C (Figure 4a). The (60)SO_3_H-UiO-66 material also maintained its crystalline structure after 5 h of reaction (Figure 4b). This fact is essential since the high chemical stability that characterizes the UiO-66 structure^43^ allows working at higher temperatures and, therefore, improves its catalytic activity concerning other MOFs such as MIL-101-SO_3_H and a commercial material such as Amberlyst-15 with a higher content of sulfonic groups but not stable in this reaction condition.^21^

The catalytic activity and stability of (60)SO_3_H-UiO-66 at 200 °C were also compared to the results of commercial acid reference heterogeneous catalysts such as Nafion-SAC-13 and Amberlyst-70 in Friedel–Crafts acylation reaction.^42,44^ Nafion-SAC-13 is an acid porous nanocomposite containing 10–20 wt % perfluorosulfonic Nafion resin on amorphous silica. This allows it to present a concentration of 0.13 mmol of S/g. Meanwhile, Amberlyst-70 is a polystyrene-based microporous ion-exchange resin comprising acidic sulfonic groups with a concentration of 2.55 mmol S/g. The catalyst loading was the same concentration as for the (60)SO_3_H-UiO-66 material, equating to 1.25 wt % of catalyst relative to the initial anisole mass. The Nafion-SAC-13 catalyst showed much higher activity (46% conversion after five h of reaction) than (60)SO_3_H-UiO-66. This result is attributed to the higher acid strength of the perfluorosulfonic groups of the Nafion-SAC-13 catalyst compared to the sulfonic groups of the (60)SO_3_H-UiO-66 sample due to the presence of fluorine atoms near the sulfonic groups.^45^ However, partial dissolution of the perfluorosulfonic catalyst was observed (only 70% of the initial catalyst was recovered after 5 h of reaction). This fact demonstrated the limited stability of the commercial Nafion-SAC-13 material at relatively high reaction temperatures. The presence of sulfur was detected in the liquid fraction of the reaction by ICP-AES analysis, confirming the dissolution of the perfluorosulfonic Nafion resin, which can also catalyze the reaction through a homogeneous catalysis process. Besides, the commercial acid Amberlyst-70 catalyst showed catalytic behavior similar to that of the Nafion-SAC-13 material, with higher anisole conversion (32%) than the MOF material. However, the catalyst was also partially dissolved as the recovered amount decreased by 50% after the reaction. It is worth noting that the selectivity value toward the p-map product remains constant at around 90% in all cases, confirming the paraselective nature of the reaction, which is independent of pore size and catalyst type, as observed in other studies.^21,46−48^

Thus, the results obtained with the sulfonic material (60)SO_3_H-UiO-66 at high reaction temperatures show that although the catalytic conversion is not as outstanding as that of both acid commercial catalysts, it is significantly more stable, whose catalytic activity only comes from the heterogeneous route, which makes it a very promising material for reactions where temperature is a significant variable as aromatic alkylation, hydration of olefins, or esterification processes.

#### 3.2.3 Stability and Reusability of the (60)SO_3_H-UiO-66 Material

**Figure 5:**
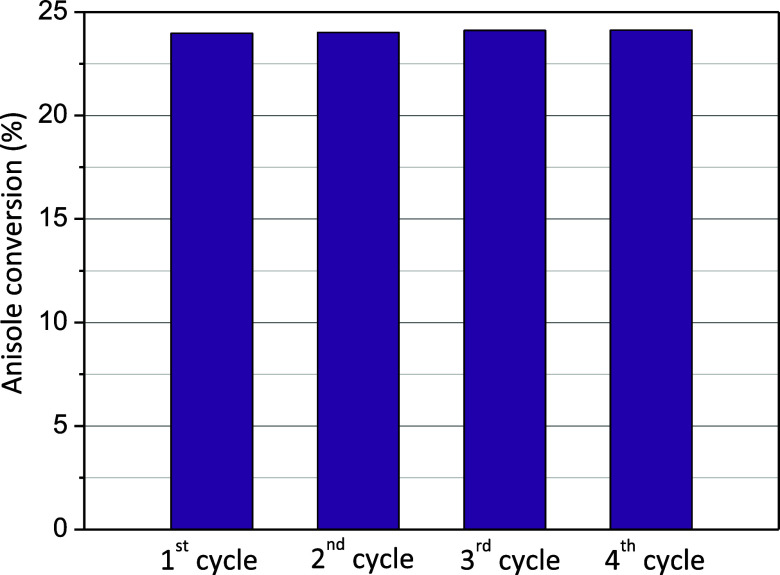
Conversion of anisole at 5 h and 200 °C using the (60)SO_3_H-UiO-66 catalyst in successive reaction cycles.

**Figure 6:**
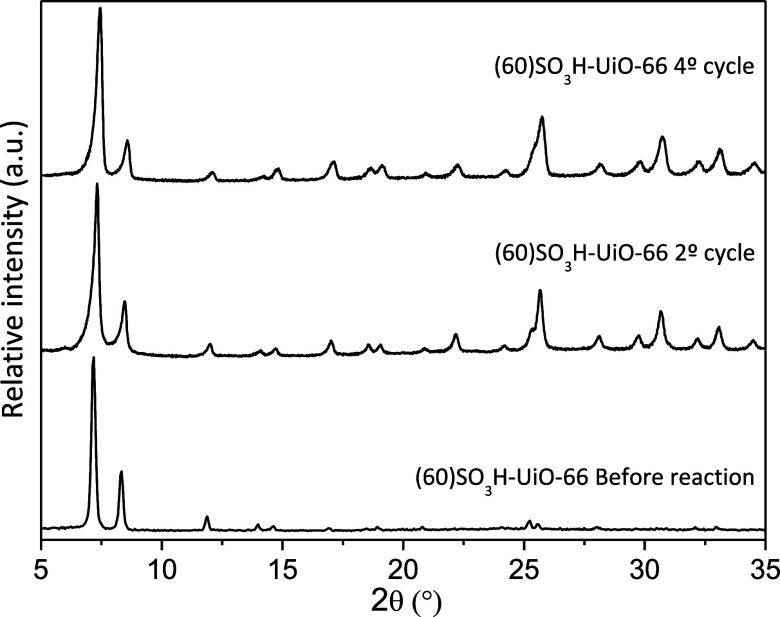
XRD patterns of the fresh and used (60)SO_3_H-UiO-66 catalyst after consecutive reactions at 200 °C.

The reusability in consecutive reaction cycles is essential when a material is used as a heterogeneous catalyst. The reusability and stability of (60)SO_3_H-UiO-66 in successive reactions as a heterogeneous catalyst were studied under the highest reaction temperature (200 °C) for four consecutive cycles using the equimolar anisole/acetic anhydride molar ratio without solvent and 1.25 wt % of catalyst relative to anisole mass. After each catalytic cycle, the catalyst was regenerated by washing with acetone and ethanol and subsequent thermal activation at 150 °C for one h under vacuum to remove the organic compounds (reactants and polyacetylated products) adsorbed on the catalyst surface. This simple regeneration method has already been evaluated for another MOF with sulfonic groups, such as MIL-101-SO_3_H, effectively removing compounds adsorbed on this material.^21^ As can be observed in Figure 5, after four consecutive reaction cycles, the (60)SO_3_H-UiO-66 material maintained constant anisole conversion near 25% and p-MAP selectivity around 92%. A slight decrease in the crystallinity of the material can be observed, but its structure is kept constant (Figure 6) and with the absence of zirconium or sulfur in the reaction medium (measured by ICP), which demonstrates the excellent stability and reusability of the prepared catalyst. HCNS also verified that sulfur content, around 1 mmol S/g, remained constant before and after the reaction. Moreover, it should be noted that the recovery of the solid catalyst was practically complete after each cycle.

#### 3.2.4 Proposed Mechanism for the Acylation of Anisole with a Sulfonic (60)SO_3_H-UiO-66 Catalyst

**Figure 7:**
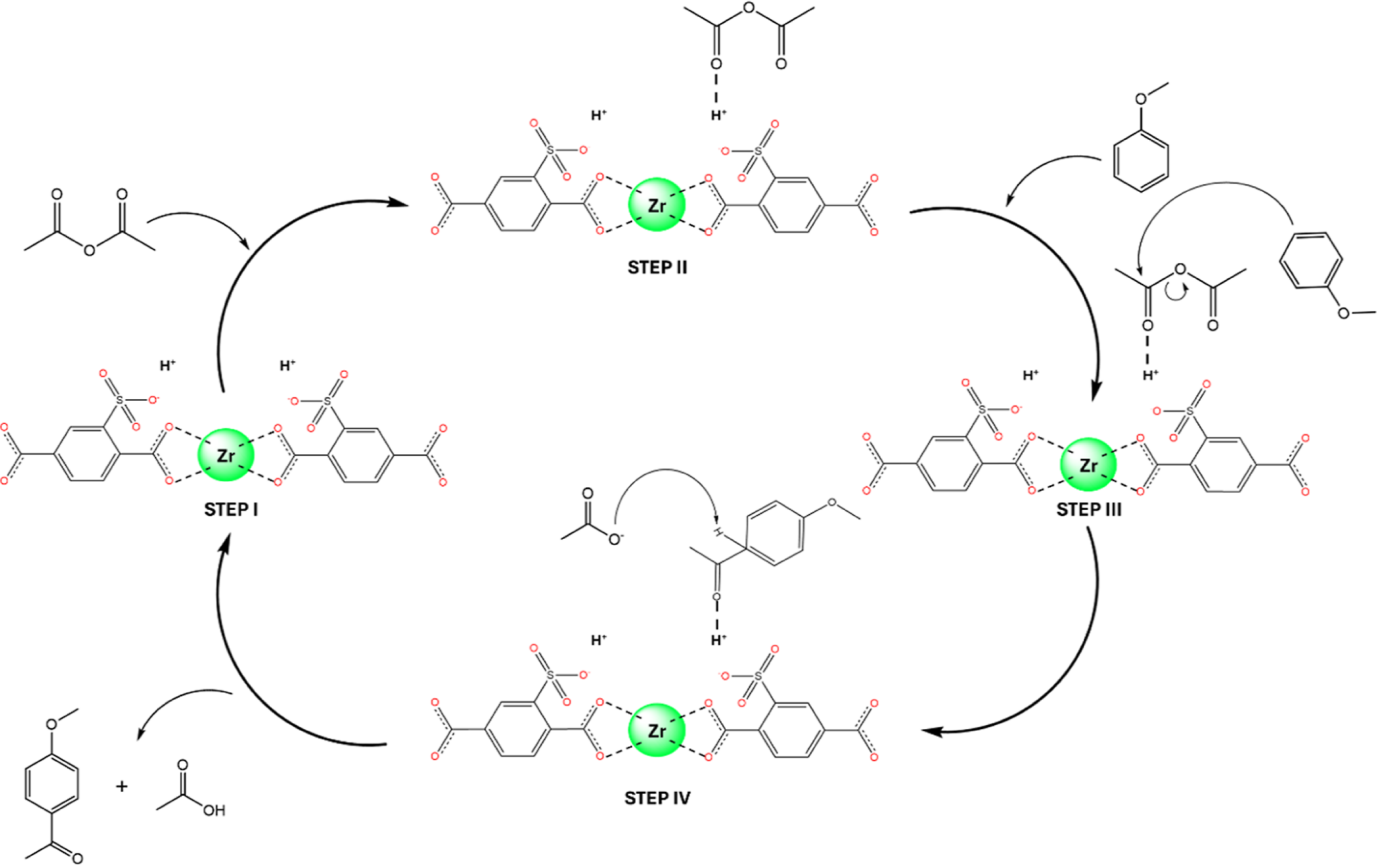
Proposed mechanism of the acylation reaction of anisole and acetic anhydride using the sulfonic UiO-66(Zr) material.

In order to investigate the mechanism of acylation of anisole with acetic anhydride using the (60)SO_3_H-UiO-66 catalyst, the nature of the acid sites present in the catalyst was examined using FTIR spectroscopy with CD_3_CN as a probe molecule (Figure S8). Adsorption of CD_3_CN revealed the predominant presence of Brønsted acid sites with different strengths, indicated by the ν(CN) bands at 2279 and 2271 cm^–1^, and a minor amount of Lewis acid sites, as shown by the ν(CN) band at 2300 cm^–1^.^49^ The band at 2260 cm^–1^ can be attributed to the physisorbed CD_3_CN. According to these results and based on previous work,^50−52^ a possible mechanism is proposed in Figure 7. In the initial stage of the process, the acetic anhydride molecule is introduced into the cycle and interacts with the H atom present in the sulfonic groups (step 2). This interaction results in the polarization of charge on C of the carbonyl group that is interacting with H^+^. This is the point at which the other molecule enters and nucleophilically attacks the charge-deficient carbonyl C of the anhydride, causing the acetate group to leave (step 3). Ultimately, in step 4, the acetate group captures the hydrogen from the aromatic ring of the anisole, forming the reaction product and acetic acid.

## 4 Conclusions

Different UiO-66(Zr) materials were synthesized by varying the sulfonic group content using a fast and sustainable microwave-assisted method. This method resulted in high synthesis yields for all synthesized materials, which exhibited the characteristic crystalline phase of UiO-66(Zr). However, an increase in the enrichment of sulfonic group ligands led to a decrease in its specific surface area due to partial pore blocking with those organic groups. The catalytic activity of sulfonic-enriched UiO-66(Zr) materials was evaluated in the acid-catalyzed Friedel–Crafts acylation of anisole with acetic anhydride. The results showed that the performance of the catalysts was dependent on the sulfonic content of the material, which was determined by the H_2_BDC–SO_3_Na/H_2_BDC molar ratio used during the synthesis process. The obtained catalytic results indicated that the higher the number of sulfonic groups in the MOF, the lower their availability due to the reduction of their textural properties; therefore, the pores were partially blocked for the reagents. The material (60)SO_3_H-UiO-66 (H_2_BDC–SO_3_Na/H_2_BDC of 60/40) demonstrated superior catalytic performance in anisole conversion among the materials tested due to a well-balanced concentration of sulfonic active sites in the opening of the porous system. This material also exhibited exceptional structural stability, enabling its use at temperatures higher than those of the commercial reference Nafion-SAC-13 and Amberlyst-70 catalysts. The remarkable stability of the heterogeneous catalyst (60)SO_3_H-UiO-66 has been demonstrated, maintaining its activity, crystallinity, and integrity during four reaction cycles at 200 °C. Additionally, it was rapidly recovered and regenerated after each cycle. The anisole conversion values and selectivity for the main products remained constant during all of the consecutive reactions.

## Supporting Information Available

Additional physicochemical characterization of all UiO-66 materials with sulfonic groups evaluated and XRD, TEM, and textural properties of the solvothermal (60)SO_3_H-UiO-66 material (PDF)

## References

[ref1] Reactions F.; Friedel C.. Kirk-Othmer Encyclopedia of Chemical Technology, Wiley, 2000; Vol. 12, p 159–198.

[ref2] Sumita A.; Ohwada T. Friedel-Crafts-Type Acylation and Amidation Reactions in Strong Brønsted Acid: Taming Superelectrophiles †. Molecules 2022, 27 (18), 5984 10.3390/molecules27185984. 36144714 PMC9503166

[ref3] Heravi M. M.; Zadsirjan V.; Saedi P.; Momeni T. Applications of Friedel-Crafts Reactions in Total Synthesis of Natural Products. RSC Adv. 2018, 8, 40061–40163. 10.1039/C8RA07325B. 35558228 PMC9091380

[ref4] Kantam M. L.; Ranganath K. V. S.; Sateesh M.; Kumar K. B. S.; Choudary B. M. Friedel-Crafts Acylation of Aromatics and Heteroaromatics by Beta Zeolite. J. Mol. Catal. A Chem. 2005, 225 (1), 15–20. 10.1016/j.molcata.2004.08.018.

[ref5] Sartori G.; Maggi R. Use of Solid Catalysts in Friedel - Crafts Acylation Reactions. Chem. Rev. 2006, 106, 1077–1104. 10.1021/cr040695c. 16522017

[ref6] Wei H.; Liu K.; Xie S.; Xin W.; Li X.; Liu S.; Xu L. Determination of Different Acid Sites in Beta Zeolite for Anisole Acylation with Acetic Anhydride. J. Catal. 2013, 307, 103–110. 10.1016/j.jcat.2013.07.010.

[ref7] Silva D. S. A.; Castelblanco W. N.; Piva D. H.; de Macedo V.; Carvalho K. T. G.; Urquieta-González E. A. Tuning the Brønsted and Lewis Acid Nature in HZSM-5 Zeolites by the Generation of Intracrystalline Mesoporosity-Catalytic Behavior for the Acylation of Anisole. Mol. Catal. 2020, 492, 111026 10.1016/j.mcat.2020.111026.

[ref8] Miao S.; Liu Y.; Zhang H.; Chang X.; Sun H.; Zhao C.; Zhang W.; Jia M. Effect of Triton X-100 Additive on the Synthesis of Beta Zeolites and Their Catalytic Application in Acylation of Anisole with Acetic Anhydride. Mater. Chem. Phys. 2022, 278, 125618 10.1016/j.matchemphys.2021.125618.

[ref9] Kaur J.; Griffin K.; Harrison B.; Kozhevnikov I. V. Friedel-Crafts Acylation Catalysed by Heteropoly Acids. J. Catal. 2002, 208 (2), 448–455. 10.1006/jcat.2002.3592.

[ref10] Kozhevnikov I. V. Friedel-Crafts Acylation and Related Reactions Catalysed by Heteropoly Acids. Appl. Catal., A 2003, 256 (1–2), 3–18. 10.1016/S0926-860X(03)00406-X.

[ref11] Cardoso L. A. M.; Alves W.; Gonzaga A. R. E.; Aguiar L. M. G.; Andrade H. M. C. Friedel-Crafts Acylation of Anisole with Acetic Anhydride over Silica-Supported Heteropolyphosphotungstic Acid (HPW/SiO2). J. Mol. Catal. A: Chem. 2004, 209 (1–2), 189–197.

[ref12] Melero J. A.; Van Grieken R.; Morales G.; Nuño V. Friedel Crafts Acylation of Aromatic Compounds over Arenesulfonic Containing Mesostructured SBA-15 Materials. Catal. Commun. 2004, 5 (3), 131–136. 10.1016/j.catcom.2003.12.007.

[ref13] Van Grieken R.; Martínez F.; Morales G.; Martín A. Nafion-Modified Large-Pore Silicas for the Catalytic Acylation of Anisole with Acetic Anhydride. Ind. Eng. Chem. Res. 2013, 52 (30), 10145–10151. 10.1021/ie401360b.

[ref14] Lee J.; Farha O. K.; Roberts J.; Scheidt K. A.; Nguyen S. T.; Hupp J. T. Metal-Organic Framework Materials as Catalysts. Chem. Soc. Rev. 2009, 38 (5), 1450–1459. 10.1039/b807080f. 19384447

[ref15] Comito R. J.; Fritzsching K. J.; Sundell B. J.; Schmidt-Rohr K.; Dincă M. Single-Site Heterogeneous Catalysts for Olefin Polymerization Enabled by Cation Exchange in a Metal-Organic Framework. J. Am. Chem. Soc. 2016, 138 (32), 10232–10237. 10.1021/jacs.6b05200. 27443860

[ref16] He W. L.; Zhao M.; Wu C. de. A Versatile Metalloporphyrinic Framework Platform for Highly Efficient Bioinspired, Photo- and Asymmetric Catalysis. Angew. Chem., Int. Ed. 2019, 58 (1), 168–172. 10.1002/anie.201810294. 30417540

[ref17] Bavykina A.; Kolobov N.; Khan I. S.; Bau J. A.; Ramirez A.; Gascon J. Metal-Organic Frameworks in Heterogeneous Catalysis: Recent Progress, New Trends, and Future Perspectives. Chem. Rev. 2020, 120 (16), 8468–8535. 10.1021/acs.chemrev.9b00685. 32223183

[ref18] Jiang J.; Gándara F.; Zhang Y. B.; Na K.; Yaghi O. M.; Klemperer W. G. Superacidity in Sulfated Metal-Organic Framework-808. J. Am. Chem. Soc. 2014, 136 (37), 12844–12847. 10.1021/ja507119n. 25157587

[ref19] Khder A. E. R. S.; Hassan H. M. A.; El-Shall M. S. Metal-Organic Frameworks with High Tungstophosphoric Acid Loading as Heterogeneous Acid Catalysts. Appl. Catal. A Gen 2014, 487, 110–118. 10.1016/j.apcata.2014.09.012.

[ref20] Hassan H. M. A.; Betiha M. A.; Mohamed S. K.; El-Sharkawy E. A.; Ahmed E. A. Stable and Recyclable MIL-101(Cr)–Ionic Liquid Based Hybrid Nanomaterials as Heterogeneous Catalyst. J. Mol. Liq. 2017, 236, 385–394. 10.1016/j.molliq.2017.04.034.

[ref21] Leo P.; Crespí N.; Palomino C.; Martín A.; Orcajo G.; Calleja G.; Martinez F. Catalytic Activity and Stability of Sulfonic-Functionalized UiO-66 and MIL-101 Materials in Friedel-Crafts Acylation Reaction. Catal. Today 2022, 390–391, 258–264. 10.1016/j.cattod.2021.10.007.

[ref22] Taddei M.; Dau P. v.; Cohen S. M.; Ranocchiari M.; van Bokhoven J. A.; Costantino F.; Sabatini S.; Vivani R. Efficient Microwave Assisted Synthesis of Metal-Organic Framework UiO-66: Optimization and Scale Up. Dalton Trans. 2015, 44 (31), 14019–14026. 10.1039/C5DT01838B. 26165508

[ref23] Klinowski J.; Almeida Paz F. A.; Silva P.; Rocha J. Microwave-Assisted Synthesis of Metal-Organic Frameworks. Dalton Trans. 2011, 40, 321–330. 10.1039/C0DT00708K. 20963251

[ref24] Chen C.; Feng X.; Zhu Q.; Dong R.; Yang R.; Cheng Y.; He C. Microwave-Assisted Rapid Synthesis of Well-Shaped MOF-74 (Ni) for CO2 Efficient Capture. Inorg. Chem. 2019, 58 (4), 2717–2728. 10.1021/acs.inorgchem.8b03271. 30720271

[ref25] Solís R. R.; Gómez-Avilés A.; Belver C.; Rodriguez J. J.; Bedia J. Microwave-Assisted Synthesis of NH2-MIL-125(Ti) for the Solar Photocatalytic Degradation of Aqueous Emerging Pollutants in Batch and Continuous Tests. J. Environ. Chem. Eng. 2021, 9 (5), 106230 10.1016/j.jece.2021.106230.

[ref26] Wang Y.; Ge S.; Cheng W.; Hu Z.; Shao Q.; Wang X.; Lin J.; Dong M.; Wang J.; Guo Z. Microwave Hydrothermally Synthesized Metal-Organic Framework-5 Derived C-Doped ZnO with Enhanced Photocatalytic Degradation of Rhodamine B. Langmuir 2020, 36 (33), 9658–9667. 10.1021/acs.langmuir.0c00395. 32787068

[ref27] Dong Y.; Hu T.; Pudukudy M.; Su H.; Jiang L.; Shan S.; Jia Q. Influence of Microwave-Assisted Synthesis on the Structural and Textural Properties of Mesoporous MIL-101(Fe) and NH2-MIL-101(Fe) for Enhanced Tetracycline Adsorption. Mater. Chem. Phys. 2020, 251, 123060 10.1016/j.matchemphys.2020.123060.

[ref28] Calleja G.; Sanz R.; Orcajo G.; Briones D.; Leo P.; Martínez F. Copper-Based MOF-74 Material as Effective Acid Catalyst in Friedel-Crafts Acylation of Anisole. Catal. Today 2014, 227, 130–137. 10.1016/j.cattod.2013.11.062.

[ref29] Biswas S.; Zhang J.; Li Z.; Liu Y.-Y.; Grzywa M.; Sun L.; Volkmer D.; van der Voort P. Enhanced Selectivity of CO2 over CH4 in Sulphonate-Carboxylate- and Iodo-Functionalized UiO-66 Frameworks. Dalton Trans. 2013, 42 (13), 4730–4737. 10.1039/c3dt32288b. 23361454

[ref30] Kandiah M.; Nilsen M. H.; Usseglio S.; Jakobsen S.; Olsbye U.; Tilset M.; Larabi C.; Quadrelli E. A.; Bonino F.; Lillerud K. P. Synthesis and Stability of Tagged UiO-66 Zr-MOFs. Chem. Mater. 2010, 22 (24), 6632–6640. 10.1021/cm102601v.

[ref31] Liang W.; Coghlan C. J.; Ragon F.; Rubio-Martinez M.; D’Alessandro D. M.; Babarao R. Defect Engineering of UiO-66 for CO 2 and H 2 O Uptake – a Combined Experimental and Simulation Study. Dalton Trans. 2016, 45 (11), 4496–4500. 10.1039/C6DT00189K. 26875692

[ref32] Jiang H.; Xue C.; Sun W.; Gong Z.; Yuan X. Acid Regulation of Defective Sulfonic-Acid-Functionalized UiO-66 in the Esterification of Cyclohexene with Formic Acid. Catal. Lett. 2023, 153 (3), 836–849. 10.1007/s10562-022-04028-w.

[ref33] Azhar M. R.; Abid H. R.; Sun H.; Periasamy V.; Tadé M. O.; Wang S. One-Pot Synthesis of Binary Metal Organic Frameworks (HKUST-1 and UiO-66) for Enhanced Adsorptive Removal of Water Contaminants. J. Colloid Interface Sci. 2017, 490, 685–694. 10.1016/j.jcis.2016.11.100. 27940035

[ref34] Devarajan N.; Suresh P. MIL-101-SO3H Metal-Organic Framework as a Brønsted Acid Catalyst in Hantzsch Reaction: An Efficient and Sustainable Methodology for One-Pot Synthesis of 1,4-Dihydropyridine. New J. Chem. 2019, 43 (17), 6806–6814. 10.1039/C9NJ00990F.

[ref35] Zhao K.; Xiang Y.; Sun X.; Chen L.; Xiao J.; Liu X. Highly Efficient One-Step Conversion of Fructose to Biofuel 5-Ethoxymethylfurfural Using a UIO-66-SO3H Catalyst. Front Chem. 2022, 10, 1–8. 10.3389/fchem.2022.900482. PMC9125248 35615317

[ref36] Mirhosseini-Eshkevari B.; Esnaashari M.; Ghasemzadeh M. A. Novel Brönsted Acidic Ionic Liquids Confined in UiO-66 Nanocages for the Synthesis of Dihydropyrido[2,3- d ]Pyrimidine Derivatives under Solvent-Free Conditions. ACS Omega 2019, 4 (6), 10548–10557. 10.1021/acsomega.9b00178. 31460153 PMC6648245

[ref37] Xu T.; Shehzad M. A.; Wang X.; Wu B.; Ge L.; Xu T. Engineering Leaf-Like UiO-66-SO3H Membranes for Selective Transport of Cations. Nanomicro Lett. 2020, 12 (1), 51 10.1007/s40820-020-0386-6. 34138245 PMC7770750

[ref38] Andriamitantsoa R. S.; Wang J.; Dong W.; Gao H.; Wang G. SO _3_ H-Functionalized Metal Organic Frameworks: An Efficient Heterogeneous Catalyst for the Synthesis of Quinoxaline and Derivatives. RSC Adv. 2016, 6 (41), 35135–35143. 10.1039/C6RA02575G.

[ref39] Sarsani V. S. R.; Lyon C. J.; Hutchenson K. W.; Harmer M. A.; Subramaniam B. Continuous Acylation of Anisole by Acetic Anhydride in Mesoporous Solid Acid Catalysts: Reaction Media Effects on Catalyst Deactivation. J. Catal. 2007, 245 (1), 184–190. 10.1016/j.jcat.2006.10.001.

[ref40] Thomas-Hillman I.; Laybourn A.; Dodds C.; Kingman S. W. Realising the Environmental Benefits of Metal–Organic Frameworks: Recent Advances in Microwave Synthesis. J. Mater. Chem. A 2018, 6 (25), 11564–11581. 10.1039/C8TA02919A.

[ref41] Phan P. T.; Hong J.; Tran N.; Le T. H. The Properties of Microwave-Assisted Synthesis of Metal–Organic Frameworks and Their Applications. Nanomaterials 2023, 13 (2), 352 10.3390/nano13020352. 36678105 PMC9864337

[ref42] Alvaro M.; Corma A.; Das D.; Fornes V.; Garcia H. Nafion”-Functionalized Mesoporous MCM-41 Silica Shows High Activity and Selectivity for Carboxylic Acid Esterification and Friedel-Crafts Acylation Reactions. J. Catal. 2005, 231 (1), 48–55. 10.1016/j.jcat.2005.01.007.

[ref43] Dhakshinamoorthy A.; Santiago-Portillo A.; Asiri A. M.; Garcia H. Engineering UiO-66 Metal Organic Framework for Heterogeneous Catalysis. ChemCatChem 2019, 11 (3), 899–923. 10.1002/cctc.201801452.

[ref44] Schuster H.; Hölderich W. F. The Acylation of 2-Methoxynaphthalene with Acetic Anhydride over Nafion/Silica Composites and BEA Zeolites Containing Lewis Acid Sites. Appl. Catal., A 2008, 350 (1), 1–5. 10.1016/j.apcata.2008.07.026.

[ref45] Siril P. F.; Davison A. D.; Randhawa J. K.; Brown D. R. Acid Strengths and Catalytic Activities of Sulfonic Acid on Polymeric and Silica Supports. J. Mol. Catal. A: Chem. 2007, 267 (1–2), 72–78. 10.1016/j.molcata.2006.11.022.

[ref46] Sharghi H.; Jokar M.; Doroodmand M. M.; Khalifeh R. Catalytic Friedel-Crafts Acylation and Benzoylation of Aromatic Compounds Using Activated Hematite as a Novel Heterogeneous Catalyst. Adv. Synth. Catal. 2010, 352 (17), 3031–3044. 10.1002/adsc.201000319.

[ref47] Gharib A.; Jahangir M.; Scheeren J. Acylation of Aromatic Compounds by Acid Anhydrides Using Preyssler’s Anion [NaP5W30O110]14- and Heteropolyacids as Green Catalysts. Pol. J. Chem. Technol. 2011, 13 (2), 11–17. 10.2478/v10026-011-0017-6.

[ref48] Freese U.; Heinrich F.; Roessner F. Acylation of Aromatic Compounds on H-Beta Zeolites. Catal. Today 1999, 49 (1–3), 237–244. 10.1016/S0920-5861(98)00429-5.

[ref49] Chakarova K.; Strauss I.; Mihaylov M.; Drenchev N.; Hadjiivanov K. Evolution of Acid and Basic Sites in UiO-66 and UiO-66-NH2Metal-Organic Frameworks: FTIR Study by Probe Molecules. Microporous Mesoporous Mater. 2019, 281, 110–122. 10.1016/j.micromeso.2019.03.006.

[ref50] Hu W. H.; Liu M. N.; Luo Q. X.; Zhang J.; Chen H.; Xu L.; Sun M.; Ma X.; Hao Q. Q. Friedel-Crafts Acylation of Anisole with Acetic Anhydride over Single- to Multiple-Layer MWW Zeolites: Catalytic Behavior and Kinetic Mechanism. Chem. Eng. J. 2023, 466, 143098 10.1016/j.cej.2023.143098.

[ref51] Desai D. S.; Yadav G. D. Friedel-Crafts Acylation of Furan Using Chromium-Exchanged Dodecatungstophosphoric Acid: Effect of Support, Mechanism and Kinetic Modelling. Clean Technol. Environ. Policy 2021, 23 (8), 2429–2441. 10.1007/s10098-021-02162-4.

[ref52] Bonati M. L. M.; Joyner R. W.; Stockenhuber M. On the Mechanism of Aromatic Acylation over Zeolites. Microporous Mesoporous Mater. 2007, 104 (1–3), 217–224. 10.1016/j.micromeso.2007.02.023.

