# Supplementary material for: Catalytic Advantages of SO3H-Modified UiO-66(Zr) Materials Obtained via Microwave Synthesis in Friedel–Crafts Acylation Reaction
Source: Inorg Chem. 2024 Sep 3;63(38):17460–8. doi: 10.1021/acs.inorgchem.4c01792 (PMC11423395; doi:10.1021/acs.inorgchem.4c01792)
Supplement: Supplementary file 1 — ic4c01792_si_001.pdf [file ic4c01792_si_001.pdf]

CATALYTIC ADVANTAGES OF SO_3H-MODIFIED UiO-66(Zr) MATERIALS OBTAINED VIA MICROWAVE SYNTHESIS IN FRIEDEL-CRAFTS ACYLATION REACTION

Marta Bauzá[a], Pedro Leo[b],* Carlos Palomino Cabello[a],* Antonio Martín[b], Gisela Orcajo[b], Gemma Turnes Palomino[a], Fernando Martínez[b,c]

[a] *Department of Chemistry, University of the Balearic Islands, Cra. de Valldemossa, 07122, Spain.*

[b] *Chemical and Environmental Engineering Group. ESCET, Universidad Rey Juan Carlos. c/Tulipán s/n 28933, Móstoles, Spain.*

[c] *Instituto de Tecnologías para la Sostenibilidad. Universidad Rey Juan Carlos. C/Tulipán s/n, 28933, Móstoles, Spain*

*To whom the correspondence should be addressed

[a*] e-mail: carlos.palomino@uib.es

[b*] e-mail: pedro.leo@urjc.es

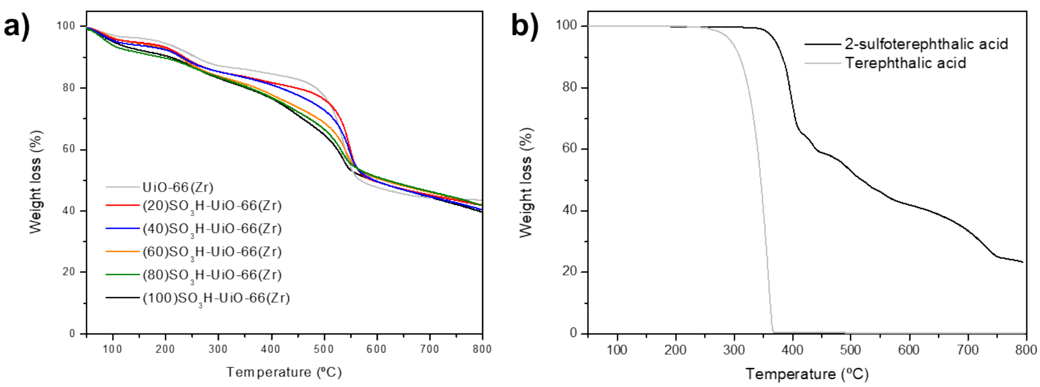

Figure S1. TGA analysis of (a) UiO-66(Zr) and SO_3H-functionalized UiO-66(Zr) samples and (b) organic linkers used in the synthesis under N_2 atmosphere.

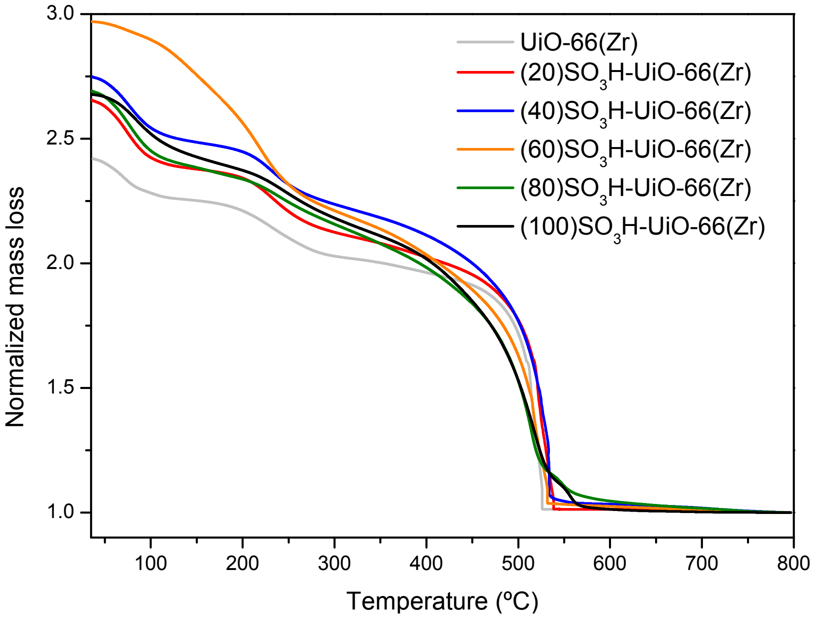

Figure S2. TGA analysis of UiO-66(Zr) and SO_3H-functionalized UiO-66(Zr) in air, normalized to the mass of the ZrO_2 decomposition product.

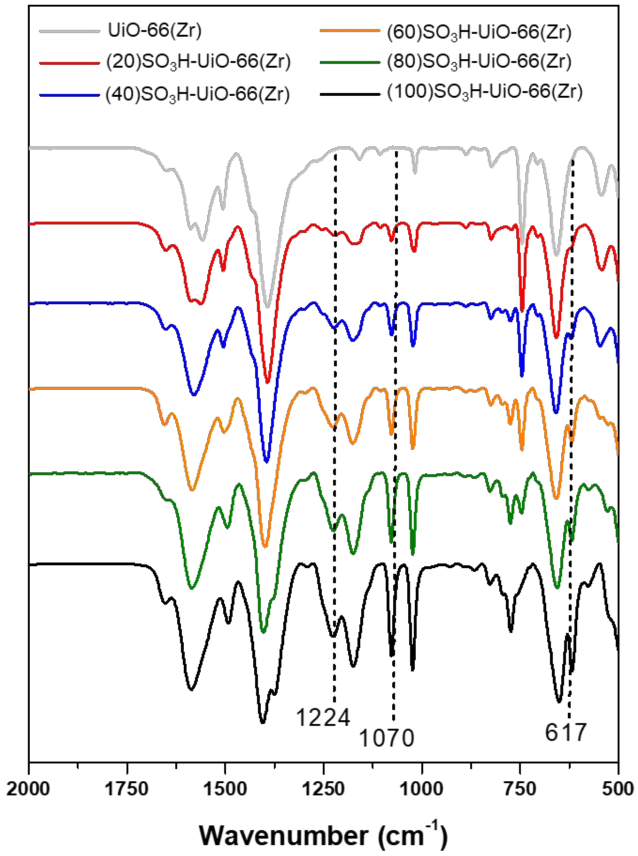

Figure S3. FTIR spectra of UiO-66(Zr) and SO_3H-functionalized UiO-66(Zr).

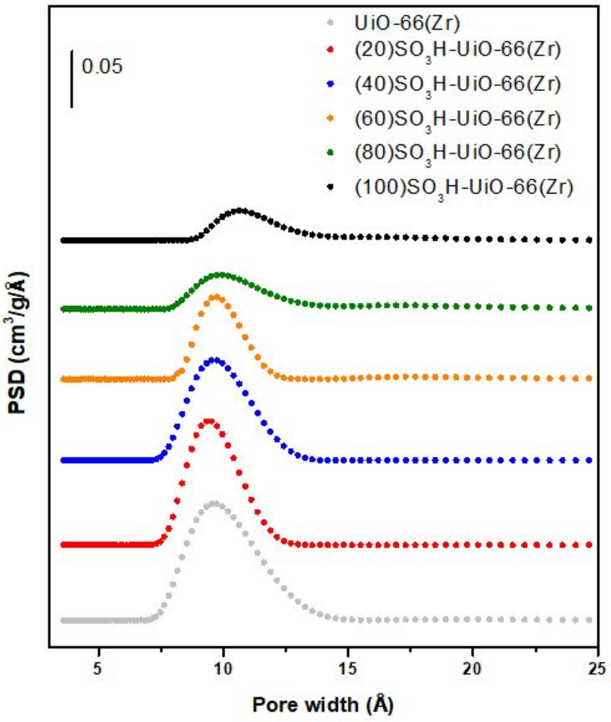

Figure S4. Pore size distribution of UiO-66(Zr) and SO_3H-functionalized UiO-66(Zr).

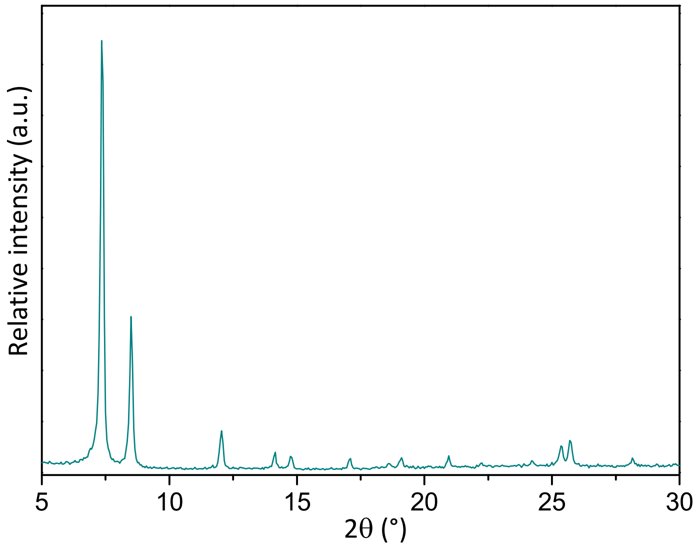

Figure S5. Experimental XRD pattern of solvothermal (60)SO_3H-UiO-66(Zr).

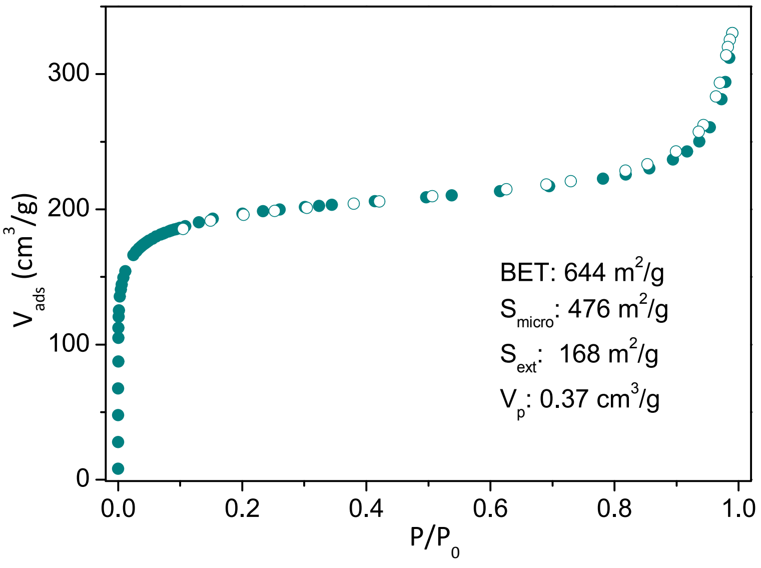

Figure S6. N_2 adsorption-desorption isotherm of solvothermal (60)SO_3H-UiO-66(Zr).

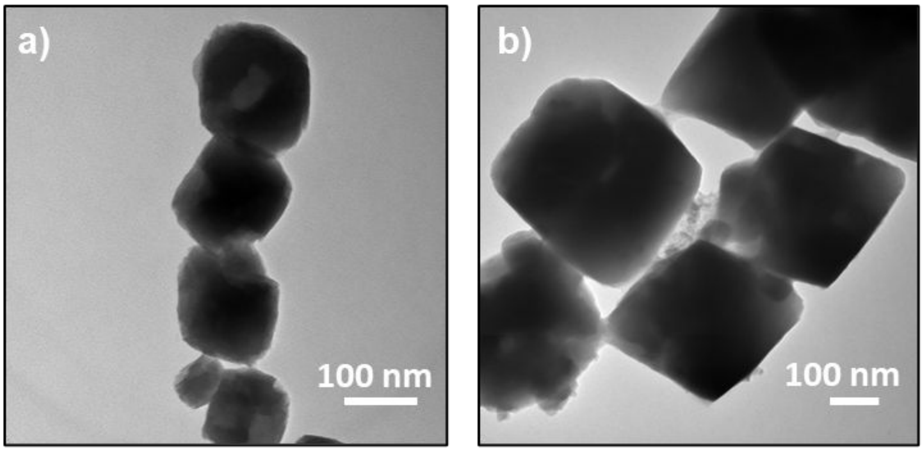

Figure S7. TEM images of (60)SO_3H-UiO-66(Zr) synthesized by (a) solvothermal and (b) microwave methods.

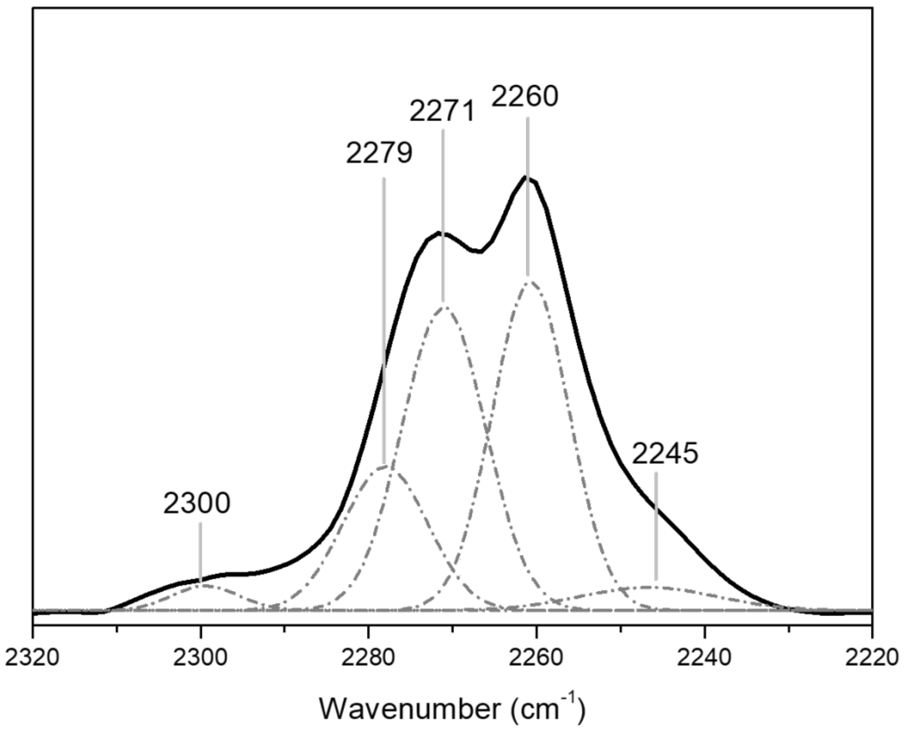

Figure S8. FTIR spectrum of CD_3CN adsorbed at room temperature of (60)SO_3H-UiO-66(Zr).
